# Efficacy and safety of Mydriatic Microdrops for Retinopathy Of Prematurity Screening (MyMiROPS): study protocol for a non-inferiority crossover randomized controlled trial

**DOI:** 10.1186/s13063-022-06243-7

**Published:** 2022-04-15

**Authors:** Aikaterini K. Seliniotaki, Anna-Bettina Haidich, Maria Lithoxopoulou, Helen Gika, Eleftheria Boutou, Christina Virgiliou, Martha Nikolaidou, Aristides Dokoumetzidis, Nikolaos Raikos, Elisavet Diamanti, Nikolaos Ziakas, Asimina Mataftsi

**Affiliations:** 1 2nd Department of Ophthalmology, School of Medicine, Faculty of Health Sciences, Aristotle University of Thessaloniki, Papageorgiou General Hospital, N.Efkarpia, 56429 Thessaloniki, Greece; 2 Department of Hygiene, Social-Preventive Medicine & Medical Statistics, School of Medicine, Faculty of Health Sciences, Aristotle University of Thessaloniki, Thessaloniki, Greece; 3 2nd Department of Neonatology, School of Medicine, Faculty of Health Sciences, Aristotle University of Thessaloniki, Thessaloniki, Greece; 4 School of Medicine, Laboratory of forensic medicine & toxicology, Aristotle University of Thessaloniki, Thessaloniki, Greece; 5 Department of Chemistry, Aristotle University of Thessaloniki, Thessaloniki, Greece; 6 Clinical Research Manager, Elpen Pharmaceutical Co.Inc., Athens, Greece; 7 Department of Pharmacy, National and Kapodistrian University of Athens, Athens, Greece

**Keywords:** Mydriasis, Phenylephrine, Preterm infants, Pupil dilation, Tropicamide

## Abstract

**Background:**

Retinopathy of prematurity (ROP) eye examination screening presupposes adequate mydriasis for an informative fundoscopy of preterm infants at risk, on a weekly basis. Systemic absorption of the instilled mydriatic regimens has been associated with various adverse events in this fragile population. This report aims to present the fully developed protocol of a full-scale trial for testing the hypothesis that the reduced mydriatic drop volume achieves adequate mydriasis while minimizing systemic adverse events.

**Methods:**

A non-inferiority crossover randomized controlled trial will be performed to study the efficacy and safety of combined phenylephrine 1.67% and tropicamide 0.33% microdrops compared with standard drops in a total of 93 preterm infants requiring ROP screening. Primary outcome will be the pupil diameter at 45 (T45) min after instillation. Pupil diameter at T90 and T120 will constitute secondary efficacy endpoints. Mixed-effects linear regression models will be developed, and the 95% confidence interval approach will be used for assessing non-inferiority. Whole blood samples will be analyzed using hydrophilic liquid chromatography–tandem mass spectrometry method (HILIC–MS/MS), for gathering pharmacokinetic (PK) data on the instilled phenylephrine, at nine specific time points within 3 h from mydriasis. Pooled PK data will be used due to ethical restrictions on having a full PK profile per infant. Heart rate, oxygen saturation, blood pressure measurements, and 48-h adverse events will also be recorded.

**Discussion:**

This protocol is designed for a study powered to assess non-inferiority of microdrops compared with standard dilating drops. If our hypothesis is confirmed, microdrops may become a useful tool in ROP screening.

**Trial registration:**

ClinicalTrials.govNCT05043077. Registered on 2 September 2021

**Supplementary Information:**

The online version contains supplementary material available at 10.1186/s13063-022-06243-7.

## Introduction

### Background and rationale

Retinopathy of prematurity (ROP) constitutes a neurovascular disorder of preterm infants and the major cause of preventable visual impairment in this population. Repetitive eye examinations, on a scheduled basis, of preterm infants who are at risk of developing ROP constitute integral part of the neonatal intensive care, worldwide [1, 2]. The ultimate goal of an efficacious ROP screening policy is to identify infants with severe disease and implement timely treatment, in an effort to reduce the unfavorable visual outcomes.

ROP screening is primarily based on an informative fundoscopy of infants at risk, prerequisite of which is the induction of adequate pupil dilation. A large variation in mydriatic regimens has been used for this purpose [3, 4], composed of either adrenergic agonists (e.g., phenylephrine) or muscarinic antagonists (e.g., tropicamide or cyclopentolate) or a combination of them, in a number of different concentrations, doses, and intervals [5, 6]. However, systemic absorption of the instilled drugs does occur and has been associated with cardiorespiratory, gastrointestinal, and central nervous system adverse events [5], in this vulnerable population of preterm infants [7].

The reduction of the instilled drop volume has been considered a promising administration technique for achieving adequate mydriasis in preterm infants, while minimizing systemic adverse events [8–11]. An external pilot crossover randomized controlled trial (RCT) has preceded, studying the effect of microdrop compared with standard drop instillation of a new proposed combination of phenylephrine 1.67% and tropicamide 0.33% that constitute routine practice in our department, while providing evidence for the feasibility of conducting a full-scale trial [12].

In the present report, we describe the fully developed protocol of the designed RCT, following the reporting guidelines of “SPIRIT 2013 Statement: Defining Standard Protocol Items for Clinical Trials” [13]. The SPIRIT checklist is presented in an additional file (see Additional file 1). The RCT has been registered in ClinicalTrials.gov (NCT05043077).

### Objectives

The primary objective of the study is to examine whether microdrops of phenylephrine 1.67% and tropicamide 0.33% are non-inferior to standard drops of the same mydriatic regimen in the induced mydriasis at 45 min after the first drop instillation (T45), in preterm infants requiring ROP screening. Secondary objectives are to examine whether microdrops are non-inferior to standard drops in the induced mydriasis at T90 and T120, in preterm infants requiring ROP screening. Secondary objectives are also to evaluate the following: (a) the pharmacokinetic profile of phenylephrine, (b) the heart rate (HR), oxygen saturation (SpO_2_), systolic (SBP), diastolic (DBP), and mean (MBP) blood pressure at T45, T90, and T120, (c) the number of hypertensive episodes during the first 24 h after mydriasis, (d) the occurrence of systemic adverse events during the 48 h after mydriasis, (e) the occurrence of local adverse events at T45, and (f) the adequacy of judging the presence or absence of treatment-requiring ROP at the end of fundoscopy, after each administration technique, in preterm infants requiring ROP screening. The *PICOS* elements of the research question are presented in Table 1.

**Table 1:** PICOS format of the research question

**Population**	Preterm infants undergoing retinopathy of prematurity screening
**Intervention**	Microdrops of phenylephrine 1.67% and tropicamide 0.33%
**Comparator**	Standard drops of phenylephrine 1.67% and tropicamide 0.33%
**Outcomes**	Efficacy: millimeters of pupil diameter Safety: occurrence of systemic adverse events and systemic absorption
**Study type**	Non-inferiority, crossover, randomized controlled trial

### Trial design

**Fig. 1:**
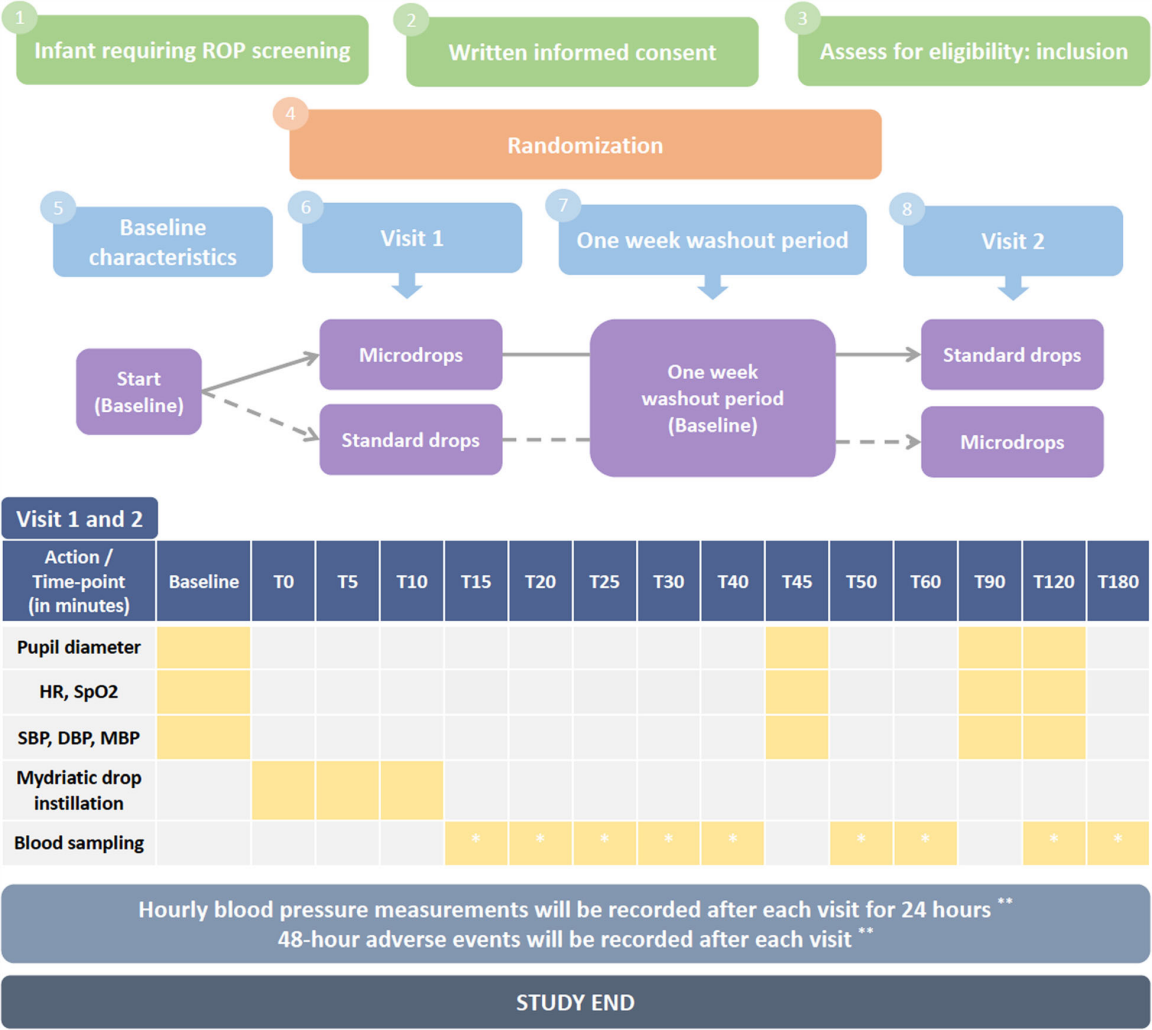
Schematic overview of the study design and the timetable of each visit. Single asterisk (*) indicates the following: each participant will be sampled once (random allocation to one time point, that would be the same for each visit). Double asterisk (**) indicates the following: available only for infants that are hospitalized in both visits. HR, heart rate; SpO_2_, oxygen saturation; SBP, systolic blood pressure; DBP, diastolic blood pressure; MBP, mean blood pressure

A non-inferiority crossover RCT is designed for answering the research question. The crossover design was chosen instead of a randomized, parallel-group design because the within-patient variation is less than the between-patient variation, thus requiring fewer patients [14]. In addition, the primary outcome does not refer to an unstable disease, and the effect of mydriasis is reversible at 2 to 3 h after instillation. Moreover, ROP screening usually necessitates weekly examinations, thus providing a sufficient washout period between the interventions. A minimum washout period of 1 week between visit 1 and visit 2 was chosen to preclude any potential carryover effect (Fig. 1). Finally, the comparator constitutes routine practice; therefore, the aim is to prove non-inferiority of the intervention, rather than superiority [15].

## Participants, interventions, and outcomes

### Study setting

The study will be conducted in a University Department of Neonatology, which includes a level IV neonatal intensive care unit (NICU), at a tertiary hospital in Northern Greece.

### Eligibility criteria

Eligible for inclusion will be preterm infants requiring ROP screening, according to the applied screening criteria (Table 2) in our department [16]. The infants will be either inpatients or outpatients, i.e., previously hospitalized and discharged before the completion of ROP screening.

**Table 2:** Applied screening criteria and screening policy in our department

**Screening criteria**	Infants with GA < 32 weeks ***and/or*** BW < 1501 g ***or*** Infants of greater GA and BW referred by the attending neonatologist due to comorbidities
**Screening commencement**	At **4–5 weeks** postnatal age for infants with GA ≥ 27 weeks At **30–31 weeks** postmenstrual age for infants with GA < 27 weeks
**Mydriasis**	Combination of phenylephrine 1.67% and tropicamide 0.33% 1 drop in each eye, for 3 doses, with 5-min intervals
**Fundoscopy**	Binocular indirect ophthalmoscopy with 28-Diopter lens, without the use of eyelid speculum or scleral indentation, performed by the same, experienced ophthalmologist (AM).

*GA* gestational age, *BW* birth weight

Exclusion criteria will include severe clinical condition with unstable vital signs precluding ophthalmological examination as judged by the attending neonatologist, congenital anomalies, clinical syndromes, severe cardiovascular disease (e.g., critical aortic stenosis, coarctation of the aorta, critical pulmonary stenosis, cardiac arrhythmias, cardiomyopathies, myocarditis [17]), inotropes’ intake during the week before enrolment, traumatic apoptosis of the corneal epithelium, corneal ulcer, and anatomical variations of the anterior segment. Infants that are outpatients at the commencement of ROP screening will also be excluded.

### Interventions

The microdrop instillation of phenylephrine 1.67% and tropicamide 0.33% will be performed using a calibrated micropipette (Nichipet EX II, 0.5–10 μL), after adjusting a disposable sterile filter tip. Microdrop volume was chosen to be 6.5 μL, which was the mean value of the range 6–7 μL of microdrops in the pilot RCT that preceded [12]. The standard drop instillation will be carried out directly through the commercially available plastic multidose dropper bottle. The standard drop volume has been measured at a range of 28–34 μL, as described in the pilot RCT [12].

The infants’ eyelids will be gently pulled open by the researcher’s (AKS) fingers, and one mydriatic drop will be instilled into each eye, with the infant in the supine position, for a total of three doses, with 5-min intervals. If the mydriatic drop falls out of the eye, it will be wiped out, and a second drop will be instilled immediately.

There are no reasons for modifying allocated interventions. Reasons for discontinuing allocated interventions (drop out criteria) include deterioration of the infant’s clinical condition that precludes eye examination or difficulties for the family to keep up with the visits for ROP screening in case of outpatients. Procedures for monitoring adherence will include phone calls for reminder a day before the appointment.

### Outcomes

Mydriatic efficacy will be defined as the millimeters (mm) of horizontal pupil diameter (right and left eye) at selected time points after the first drop instillation. The primary outcome will be the mydriatic efficacy at 45 min (T45), a time point around the time of fundoscopy. Secondary outcomes will include mydriatic efficacy at 90 (T90) and 120 (T120) min. The sustainment of mydriasis at these time points constitutes a meaningful outcome for busy departments, such as ours.

All safety outcomes will be evaluated as secondary endpoints. These will include:

- The pharmacokinetic (PK) profile of phenylephrine: area under the whole blood concentration versus time curve (AUC), maximum (peak) whole blood concentration (Cmax), time to reach maximum (peak) whole blood concentration (Tmax), and elimination half-life (T1/2). These parameters will be calculated by pooling the whole blood concentration of phenylephrine at each time point of blood sampling (T15, T20, T25, T30, T40, T50, T60, T120, T180), using both non-compartmental analysis (NCA) and model-based approaches with appropriate software for PK analysis such as Phoenix WinNonlin (Certara, Princeton, NJ). Of note, each participant will be sampled once, after random allocation to one time point, that would remain the same for each visit. The pooling of sparse PK data is considered an applicable and acceptable way to circumvent the limitations in volumes, samples, and number of observations that for ethical reasons preclude the possibility of having a full PK profile per infant.
- The mean value of HR, SpO2, SBP, DBP, and MBP at T45, T90, and T120.
- The mean number of hypertensive episodes during the first 24 h after mydriasis. We will use the same definitions as in our pilot RCT, i.e., values of SBP or DBP beyond the upper limits of 95% confidence interval according to the linear regression analysis of mean SBP and DBP by postconceptual age in weeks, by Flynn JT 2000, will be regarded as hypertension [18]. Infant’s movements or bottle feeding may interfere with hourly blood pressure measurements at the respective time points. Therefore, episodes of hypertension will be calculated as percentage of total number of recorded measurements for each individual.
- The number of participants with systemic adverse events during the 48 h after mydriasis (Table 3). The study by Mitchell et al., which showed increased apnea events during the 24–48 h period after the ROP eye examination screening, prompted us to choose this follow-up period [3].
- The number of participants with local adverse events (periorbital pallor, eyelid swelling, flushing) at T45.
- The adequacy of judging the presence or absence of treatment-requiring ROP at the end of the fundus examination (fundoscopy).

**Table 3:** Forty-eight-hour adverse events

Adverse event	Definition
Apnea	Cessation of breathing for more than 20 s, or a shorter pause accompanied by bradycardia (< 100 beats per minute) and/or oxygen desaturation [19]
Increased gastric residuals	An aspirated amount of > 2 ml/Kg or > 50% of the previous feeding volume from the stomach, following administration of enteral feeding, as evaluated in preterm infants who are being fed via an orogastric or nasogastric tube [20, 21]
Inhibited duodenal motor activity	Lower duodenal motility during fasting [22]
Delayed gastric emptying	Large gastric residual volumes associated with enteral feeding as a result of gastroparesis or delayed gastric emptying [21]
Feeding intolerance	Inability to digest enteral feedings, associated with increased gastric residuals, abdominal distension, and/or emesis [23]. If the combination of the above phenomena is observed, this will constitute diagnosis of feeding intolerance; otherwise these phenomena will be annotated individually
Abdominal distension	An actual increase in abdominal size and measurable change (≥ 2 cm) in abdominal girth [24]
Vomiting	At least one episode of uncomfortable, involuntary, forceful throwing up of gastric content, as distinguished from spitting up that occurs because of rapid infant feeding, air swallowing, or overfeeding
Paralytic ileus	Impaired motor activity of the bowel without the presence of a physical obstruction
Acute gastric dilatation	Distension of abdomen with constant nasogastric aspirate and/or absence of gas in distal bowel along with the presence of dilated stomach radiographically, which is frequently associated with feeding intolerance or sometimes is associated with a surgical condition, e.g., necrotizing enterocolitis or bowel obstruction [21, 25].
Necrotizing enterocolitis	Bell’s stage ≥ II [26]

### Participant timeline

Schematic diagram is presented in Fig. 1. We based our choice of washout period (at least 1 week) on the information that intravenous phenylephrine has an effective half-life of 5 min and an elimination half-life of 2.5 h [27]. Thus, we assume that by the end of 7 days after instillation, no drug has remained in the body. Additionally, the effect of mydriasis lasts a couple of hours; thus, a week’s interval safely separates the effect of each period’s intervention. Finally, a week’s interval respects the normal routine of weekly retinal examinations, avoiding additional stress to the infant.

### Sample size calculation

The sample size calculation was based on data from the preceded pilot RCT [12] for the primary outcome (mydriatic efficacy at T45) and was conducted using the statistical program R, version 4.0.3, and the package “PowerTOAST.” For power 90%, one-sided significance level 2.5%, mean difference in pupil diameter at T45 between microdrops and standard drops − 0.1 mm, within-subject standard deviation (SD) 0.55, and pre-defined non-inferiority margin − 0.4 mm, the calculated sample size is 74 patients. Assuming a 20% attrition rate, a sample size of 93 infants will be required. Of note, there are no previous data from reference-placebo controlled trials regarding the standard drops of the specific mydriatic regimen. Hence, the non-inferiority margin, as the minimum clinically acceptable difference, was agreed among clinical experts of the investigation team.

### Recruitment

The study setting constitutes a busy University Department of Neonatology with a level IV NICU, which enables adequate participants’ enrolment for reaching the target sample size. All infants requiring ROP screening, according to the applied ROP screening criteria in our department (Table 2) [16], will be regarded as potentially eligible, and they will be identified by the neonatologist (ML) on a weekly basis. The neonatologist (ML) will approach all these infants’ parents/guardians, who regularly visit the department to see their babies, and will recruit them after discussion during the first month of life (before the commencement of ROP screening).

## Assignment of interventions

Simple randomization, with a 1:1 allocation ratio, will be used to assign participants to the sequence of the administration technique, precluding prediction of future allocations. The unit of randomization is the infant, not the eyes. Additionally, block randomization will be used for allocating participants to one of the nine time points of blood sampling, ensuring the balance of the number of infants assigned to each time point. An independent statistician will generate these two randomization sequences separately, using a computer-generated random number table (for the simple randomization) and computer-generated permuted blocks of nine (for block randomization), and will create sequentially numbered, opaque, sealed envelopes (SNOSEs) for allocation concealment. The SNOSEs will be stored in a safe locker, accessible only by the researcher (AKS).

The neonatologist (ML) will obtain written informed consent from the parents/guardians for the infants’ enrollment, assess infants’ eligibility, and enroll participants. The researcher (AKS) will assign the enrolled participants to interventions and perform mydriasis in all cases, without participating in the enrollment or the outcome assessment and without communicating with the rest of the study team. All the study team, but the researcher (AKS), will be masked to the allocation sequence. Participants’ parents will also be masked. No reasons for unmasking will exist.

## Data collection

### Baseline characteristics

Demographics and data from the infants’ history (Table 4) will be collected from the infants’ medical records, by the neonatologist (ML). Eye color (light, dark) will be determined upon initial assessment of the pupil diameter at T0. Pupil diameter, HR, SpO2, SBP, DBP, and MBP values will be measured and recorded by the outcome assessor (AM, ML) at T0.

**Table 4:** Demographics and data from the infants’ history

Demographics	Infant’s history ^a^
Gender	Maternal hypertension
Ethnicity	Antenatal steroids
Gestational age (in weeks)	Postnatal acute renal failure
Birth weight (in grams)	Bronchopulmonary dysplasia
Postmenstrual age (in weeks)	Patent ductus arteriosus
Weight (in grams)	Necrotizing enterocolitis
	Sepsis^b^
	Days with indwelling blood vessel catheters^c^

^a^Comorbidities that constitute potential risk factors for neonatal hypertension [28]

^b^Defined as one positive blood culture with a clinical picture suggestive of systemic infection

^c^Umbilical arterial catheter (UAC) or umbilical venous catheter (UVC) or peripherally inserted central catheter (PICC) line

### Mydriatic efficacy

The infants’ eyelids will be gently pulled open by the assessor’s fingers, and the pupil diameter will be measured using the brightest illumination from the indirect ophthalmoscope and a customized ruler in 0.5 mm increments as described in the pilot RCT [12]. When indecisive between two consecutive increments, the assessor will choose the smaller value, adopting a conservative approach. In the pilot RCT, the two outcome assessors (AM, ML) presented agreement in their measurements (intraclass correlation coefficient range: 0.87 to 0.97) [12]; therefore, one of them (whoever is available) will assess mydriatic efficacy in the present RCT. For each participant, the mean value of pupil diameter between left and right eye will be used for the analyses.

### Safety outcomes

The HR (beats per minute), SpO_2_ (%), SBP, DBP, and MBP (mmHg) values will be recorded by the outcome assessor as measured using a connected monitor (Draeger®), a disposable SpO_2_ sensor (Masimo®) on the foot of the infant and a disposable blood pressure cuff with an appropriate size covering two thirds of the upper arm, as was the case in our pilot RCT [12]. Hourly blood pressure measurements will be taken automatically by the connected monitor for the first 24 h after each visit. The 48-h adverse events will be assessed and recorded by the care providers (independent masked observers). Apart from the listed potential 48-h adverse events (Table 3), any others that may occur will also be recorded on the CRF.

Blood sampling for PK analysis will be combined with blood sampling for routine examinations from the infant’s peripheral vessel, for the avoidance of additional painful procedures. The post-mydriasis in-house blood samples will be collected within 1 min from the scheduled sampling time for the samples drawn until Τ60 and within 5 min for samples drawn at T120 and T180. The exact time of blood collection will be recorded. Non-nutritive sucking with oral glucose will be used for pain-relief some minutes before the blood sampling. A heparinized 2.5 ml syringe will be used to collect 0.25 ml peripheral whole blood that will directly be stored in a pre-labeled microtainer blood collection tube with EDTA in − 80 °C, until analysis.

The whole blood will be analyzed within a standardized period of 2 months. Phenylephrine blood concentrations will be determined by a liquid chromatography–tandem mass spectrometry (LC-MS/MS) method. Analysis will be performed on an ACQUITY UPLC - Xevo TQD system (Waters, UK). Hydrophilic interaction liquid chromatography (HILIC) will be performed on an ACQUITY UPLC BEH AMIDE column (2.1 × 150 mm, 1.7 μm), under isocratic elution conditions with acetonitrile–water (88.5:11.5, v/v), 0.1% formic acid, (pH 3) at a 0.5 ml/min flow rate, and at 40 °C column’s temperature. Positive electrospray ionization mode will be applied, and the detection will be performed by monitoring the MRM transition of m/z 168 > 150. Etilefrine will be used as internal standard (IS). In 50 μL of the samples, 20 μL of IS solution and then 130 μL of acetonitrile–water (95:5, v/v) will be added. The mixture will be vortexed and then centrifuged 10 min at 10000 rpm. From the supernatant, 10 μL will then be directly injected into the LC-MS/MS system. The method was validated and showed accuracy with recovery (%) ranging from 90 to 92% and precision with RSD (%) from 2 to 10% within a linear range of 1–20 ng/ml in blood. Stability of the drug in blood was found to be within acceptable limits under − 80 °C for 2 months.

### Data management

Paper case report forms (CRF) will be used, accompanied by a data dictionary. CRFs have been pilot tested during the pilot RCT. Each participant will be represented by a unique identification number (ID), and all data will be collected anonymously. All CRFs will be stored in a safe locker, and a complete digital backup (scanning of the original CRFs) will be performed twice a month by the outcome assessors (AM, ML).

Source data verification (SDV) will be implemented through bimonthly on-site monitoring visits by independent personnel. This personnel will verify selected crucial aspects of data, including eligibility criteria, informed consent form, demographic characteristics, and adverse events, for a random 25% of randomized participants.

The data on the CRFs will be transferred to an *Excel* spreadsheet. Cross check of all data on the *Excel* against the CRFs will be performed by independent personnel before the analysis. Any discrepancies will be corrected according to the original data.

## Statistical analysis plan

Data analysis will be conducted using the statistical program R, version 4.0.3. Reporting of the results will follow the “*Reporting Noninferiority and Equivalence Randomized Trials: Extension of the Consolidated Standards of Reporting Trials (CONSORT) 2010 statement*” [29].

The dataset will comprise all participants who were randomized and received at least one of the mydriasis techniques after having recorded the baseline characteristics. Participants discontinuing the trial during the first period or during the washout period will be excluded (full analysis set). We will report reasons for withdrawal for each allocation sequence group and compare the reasons qualitatively.

The assumption of normal distribution will be investigated for all continuous variables (GA, BW, PMA, weight, days with indwelling catheters, percentage of hypertensive episodes per number of recordings) using Shapiro-Wilk test, histograms, and statistical moments (skewness and kurtosis).

### Baseline characteristics

Demographics and data from the infants’ history will be presented separately for each mydriasis technique sequence (microdrops first: M/S group and standard drops first: S/M group). If data are normally distributed, they will be represented with mean and SD. Otherwise, median and interquartile range (IQR) will be generated for skewed data. Categorical variables including gender, ethnicity, eye color, and the number of comorbidities (maternal hypertension, antenatal steroids, postnatal acute renal failure, bronchopulmonary dysplasia, patent ductus arteriosus, necrotizing enterocolitis, sepsis) will be summarized with frequencies and percentages.

### Efficacy outcomes

The primary analysis will follow the intention-to-treat (ITT) principle. As sensitivity analyses, we will use the full analysis set for our primary outcome analysis after excluding cases with missing primary outcome values and the per-protocol (PP) analysis set, as more conservative in non-inferiority trials [30]. In PP analysis, participants will be analyzed according to the mydriasis technique that they received. Only patients who satisfy eligibility criteria and properly follow the protocol will be included in PP analysis. The application of washout period shorter than 1 week and the potential need for additional mydriatic drop instillation will be considered as protocol violations. Multiple imputation will be used for handling missing data, using the chained equation approach [31]. In a sensitivity analysis, the results of the complete case analysis of the primary outcome will be compared with multiple imputation methodology to assess the robustness of our findings.

**Fig. 2:**
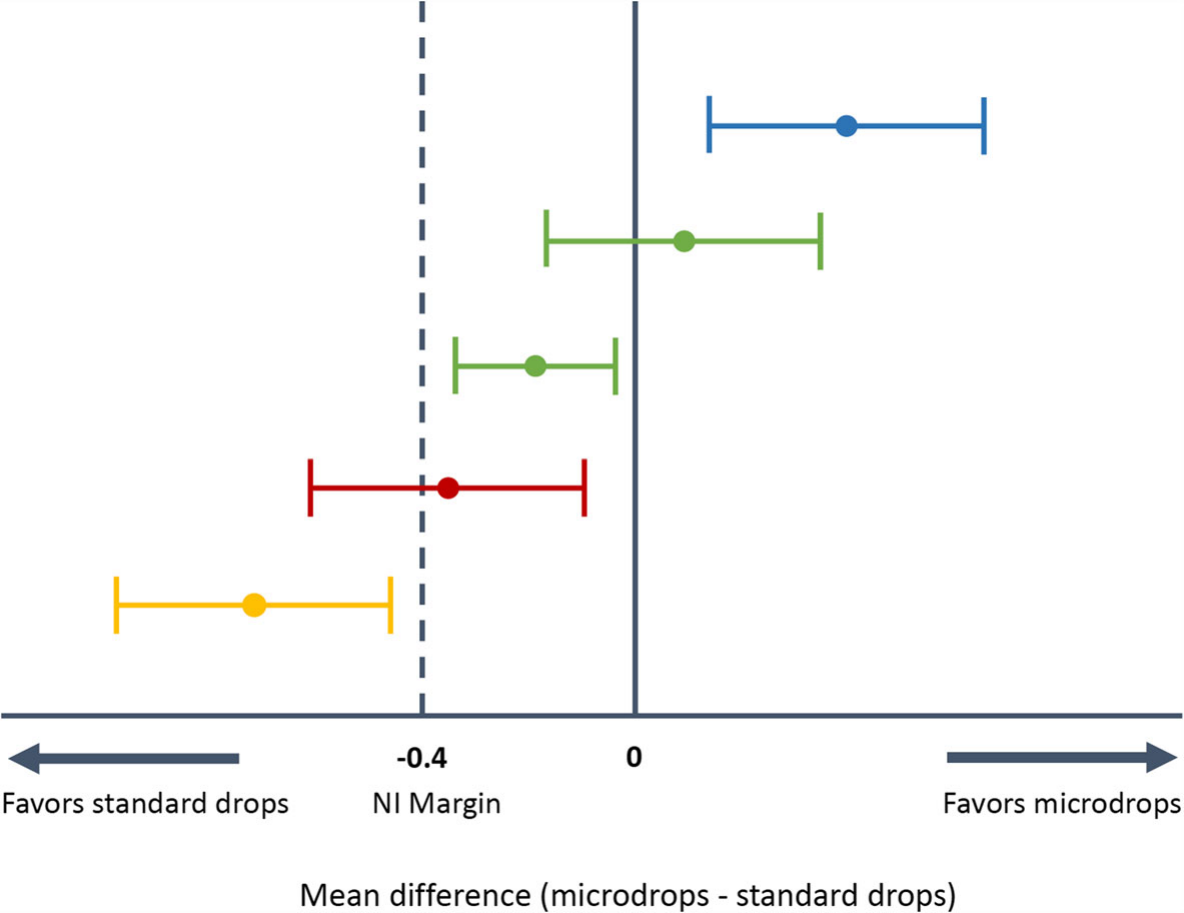
Interpretation of possible results. Green: Non-inferiority (of microdrops versus standard drops) shown. Red: Non-inferiority not shown (inconclusive trial). Blue: Superiority shown. Yellow: Inferiority shown. NI margin: non-inferiority margin

The primary outcome and all secondary efficacy outcomes will be analyzed using a linear regression mixed-effects model to adjust for the crossover design, including as fixed effects the instillation method, the period, the sequence, the pupil diameter at T0, and as random effect the participant ID. Adjustment of multiplicity will be performed using Bonferroni correction. Non-inferiority of microdrops compared with standard drops will be judged based on the 95% confidence intervals (CI) obtained from the mixed-effects models. When the lower limit of the 95% CI falls above the predefined non-inferiority margin, non-inferiority will be concluded. The interpretations of all possible results based on the 95% CI approach are displayed in Fig. 2.

### Safety outcomes

We will apply a linear regression mixed-effects model for the continuous variables of HR, SpO_2_, SBP, DBP, and MBP values at T45, T90, and T120 between standard drops and microdrops to adjust for the cross-over design. No multiplicity adjustments will be performed due to the exploratory nature of the safety outcomes. Each 48-h adverse event will be analyzed separately. The number of systemic adverse events during 48 h after mydriasis will be compared between the two administration techniques with McNemar’s test or the binomial exact test. In case a substantial number of adverse events are observed, a mixed-effects logistic regression model will be performed on the proportion of patients with systemic adverse events separately or as a composite outcome. The instillation method, the period, and the sequence will be used as fixed effects, and the participant ID will be used as random effect. The parametric paired *t*-test or the non-parametric Wilcoxon signed-rank test will be used to compare the percentage of hypertensive episodes per number of recordings between the two administration techniques.

For all analyses, the significant level will be set at 5%, and two-tailed tests will be performed.

### PK analysis

The pooled PK data will be illustrated as a scatterplot with an average line which will mirror the average blood concentration of phenylephrine in all tested infants at that time point.

### Subgroup analyses

Subgroup analyses will be performed according to GA, i.e., GA < 28 weeks and GA ≥ 28 weeks. If the available number of data permits, subgroup analyses will also be performed according to the stage of ROP.

### Interim analyses and stopping guidelines

There will be no interim analysis of efficacy or safety in this trial and no stopping guidelines will exist.

## Data monitoring

In the present study, a Trial Steering Committee or a Data Monitoring Committee will not be appointed, and no auditing has been planned, as this is a low-risk trial.

## Ethics and dissemination

All procedures will be in accordance with the 1964 Helsinki declaration and its later amendments. The study has been approved by the Institutional Review Board (IRB) of the Medical School of Aristotle University of Thessaloniki, and by the Bioethics Committee of Papageorgiou General Hospital of Thessaloniki. Written informed consent will be obtained from the parents/guardians for the infant’s enrollment.

### Protocol amendments

Any protocol amendments will be submitted to the IRBs for approval. For future protocol modifications, the date of the amendment, a description of the changes and the rationale will be publicly available, in an Appendix. Minor amendments that have been applied so far are presented in an additional file (see Additional file 2).

### Ancillary and post-trial care

Inpatients will receive all required tests and treatments, should any adverse event occur. Similarly, families whose infants have been recently discharged from the NICU have a follow-up plan in outpatients and also enjoy receiving access to the department’s services in case of adverse events before their follow-up appointment. Completion of ROP screening is provided to all infants post-trial, and any required treatment or follow-up is scheduled appropriately.

### Dissemination policy

The trial results will be presented at an international conference and published in a peer-review journal.

## Trial status

Recruitment status: Recruiting

Protocol version number and date: Version 1.2, February 24, 2022

Date recruitment began: September 7, 2021

Approximate date when recruitment will be completed: December 15, 2022

## Supplementary Information

**Additional file 1.**

**Additional file 2.**

## Data Availability

Deidentified individual participant data (IPD) that underline published results, along with related data dictionaries, will be available from 3 months to 36 months following results’ publication, only to researchers who will provide a methodologically sound proposal, for types of analyses to achieve aims in the approved proposal or for individual participant data meta-analysis, and only after acceptance of the proposed protocol by our Institution’s IRB. Proposals should be directed to the corresponding author (AM) and data requestors will need to sign a data access agreement. The study protocol and statistical analysis plan will also be available, if needed.

## Abbreviations

BW: Birth weight
CI: Confidence Interval
CRF: Case report form
DBP: Diastolic blood pressure
GA: Gestational age
HR: Heart rate
ITT: Intention to treat
IQR: Interquartile range
LC-MS/MS: Liquid chromatography–tandem mass spectrometry
MBP: Mean blood pressure
NICU: Neonatal intensive care unit
PK: Pharmacokinetic
RCT: Randomized controlled trial
ROP: Retinopathy of prematurity
SBP: Systolic blood pressure
SD: Standard deviation
SNOSE: Sequentially numbered opaque sealed envelope
SpO2: Oxygen saturation
PP: Per protocol

## References

[1] Hellström A, Smith LEH, Dammann O (2013). Retinopathy of prematurity. Lancet..

[2] Fierson WM (2013). Screening examination of premature infants for retinopathy of prematurity. Pediatrics..

[3] Mitchell AJ, Green A, Jeffs DA, Paula K, Roberson P (2011). Physiologic effects of retinopathy of prematurity screening examinations. Adv Neonatal Care.

[4] Lux AL, Degoumois A, Barjol A, Mouriaux F, Denion E (2017). Combination of 5% phenylephrine and 0.5% tropicamide eyedrops for pupil dilation in neonates is twice as effective as 0.5% tropicamide eyedrops alone. Acta Ophthalmol.

[5] Kremer LJ, Reith DM, Medlicott N, Broadbent R (2019). Systematic review of mydriatics used for screening of retinopathy in premature infants. BMJ Paediatr Open.

[6] Seliniotaki AK, Prousali E, Lithoxopoulou M, Kokkali S, Ziakas N, Soubasi V, Mataftsi A (2020). Alternative mydriasis techniques for retinopathy of prematurity screening. Int Ophthalmol.

[7] Alcorn J, McNamara PJ (2003). Pharmacokinetics in the newborn. Adv Drug Deliv Rev.

[8] Lynch MG, Brown RH, Goode SM, Schoenwald RD, Chien S (1987). Reduction of phenylephrine drop size in infants achieves equal dilation with decreased systemic absorption. Arch Ophthalmol.

[9] Wheatcroft S, Sharma A, McAllister J (1993). Reduction in mydriatic drop size in premature infants. Br J Ophthalmol.

[10] Elibol O, Alçelik T, Yüksel N, Çaǧlar Y (1997). The influence of drop size of cyclopentolate, phenylephrine and tropicamide on pupil dilatation and systemic side effects in infants. Acta Ophthalmol Scand.

[11] Kremer LJ, Broadbent R, Medlicott N, Sime MJ, McCaffrey F, Reith DM (2021). Randomised controlled pilot trial comparing low dose and very low- dose microdrop administration of phenylephrine and cyclopentolate for retinopathy of prematurity eye examinations in neonates. Arch Dis Child.

[12] Seliniotaki AK, Lithoxopoulou M, Talimtzi P, Georgiou E, Diamanti E, Ziakas N, Haidich AB, Mataftsi A (2022). Efficacy and safety of mydriatic microdrops for retinopathy of prematurity screening: an external pilot crossover randomized controlled trial. J Perinatol.

[13] Chan AW, Tetzlaff JM, Altman DG, Laupacis A, Gøtzsche PC, Krleža-Jerić K, Hróbjartsson A, Mann H, Dickersin K, Berlin JA, Doré CJ, Parulekar WR, Summerskill WSM, Groves T, Schulz KF, Sox HC, Rockhold FW, Rennie D, Moher D (2013). SPIRIT 2013 statement: defining standard protocol items for clinical trials. Chin J Evid Based Med.

[14] Pocock SJ. Clinical trials: a practical approach: Wiley; 2013. 10.1002/9781118793916.

[15] Walker J (2019). Non-inferiority statistics and equivalence studies. BJA Educ.

[16] Mataftsi A, Moutzouri S, Karagianni P, Ziakas N, Soubasi V, Brazitikos P, Haidich AB (2020). Retinopathy of prematurity occurrence and evaluation of screening policy in a large tertiary Greek cohort. Int Ophthalmol.

[17] Mellander M (2013). Diagnosis and management of life-threatening cardiac malformations in the newborn. Semin Fetal Neonatal Med.

[18] Flynn JT (2000). Neonatal hypertension: diagnosis and management. Pediatr Nephrol.

[19] Eichenwald EC. Apnea of prematurity. Pediatrics. 2016;137(1). 10.1542/peds.2015-3757. 10.1542/peds.2015-3757 26628729

[20] Li YF, Lin HC, Torrazza RM, Parker L, Talaga E, Neu J (2014). Gastric residual evaluation in preterm neonates: a useful monitoring technique or a hindrance?. Pediatr Neonatol.

[21] Abiramalatha T, Thanigainathan S, Ninan B (2019). Routine monitoring of gastric residual for prevention of necrotising enterocolitis in preterm infants. Cochrane Database Syst Rev.

[22] Bonthala S, Sparks JW, Musgrove KH, Berseth CL (2000). Mydriatics slow gastric emptying in preterm infants. J Pediatr.

[23] F Fanaro S (2013). Feeding intolerance in the preterm infant. Early Hum Dev.

[24] Lucchini R, Bizzarri B, Giampietro S, De Curtis M (2011). Feeding intolerance in preterm infants. How to understand the warning signs. J Matern Neonatal Med.

[25] Sarıcı SÜ, Yurdakök M, Ünal Ş (2001). Acute gastric dilatation complicating the use of mydriatics in a preterm newborn. Pediatr Radiol.

[26] Bell MJ, Ternberg JL, Feigin RD, Keating JP, Marshall R, Barton L (1978). Neonatal necrotizing enterocolitis. Therapeutic decisions based upon clinical staging. Ann Surg.

[27] FDA Approved drug products: phenylephrine intravenous injection (ready to use). https://www.accessdata.fda.gov/drugsatfda_docs/label/2019/212909s000lbl.pdf. Accessed 20 Feb 2022.

[28] Dionne JM, Abitbol CL, Flynn JT (2012). Hypertension in infancy: diagnosis, management and outcome. Pediatr Nephrol.

[29] Piaggio G, Elbourne DR, Pocock SJ, Evans SJW, Altman DG (2012). Reporting of noninferiority and equivalence randomized trials: extension of the CONSORT 2010 statement. JAMA.

[30] D’Agostino RB, Massaro JM, Sullivan LM (2003). Non-inferiority trials: design concepts and issues - the encounters of academic consultants in statistics. Stat Med.

[31] Horton NJ, Kleinman KP (2007). Much ado about nothing: a comparison of missing data methods and software to fit incomplete data regression models. Am Stat.

