# Supplementary material for: Efficacy and safety of Mydriatic Microdrops for Retinopathy Of Prematurity Screening (MyMiROPS): study protocol for a non-inferiority crossover randomized controlled trial
Source: Trials. 2022 Apr 15;23:322. doi: 10.1186/s13063-022-06243-7 (PMC9013111; doi:10.1186/s13063-022-06243-7)
Supplement: Supplementary file 2 — Additional file 2. [file 13063_2022_6243_MOESM2_ESM.docx]

**SUPPLEMENTARY MATERIAL**

**Table**: List of protocol amendments so far.

| **Protocol**  **modification** | **Date of**  **amendment** | **Description**  **of changes** | **Rationale** |
| --- | --- | --- | --- |
| Additional exclusion criterion | 14/10/2021 | Infants that are outpatients at the commencement of ROP screening will be excluded. ^*^ | This additional exclusion criterion was decided due to difficulties in collecting the safety outcomes in infants that are outpatients in both Visits. |
| IPD sharing statement | 14/10/2021 | Plan to share IPD was scheduled, as described within the protocol. | To comply with the requirements of the ICMJE. |
| Ancillary and post-trial care statement | 24/2/2022 | Ancillary and  post-trial care was detailed in the protocol. | Upon reviewer’s suggestion. |
| Statistical analysis plan regarding baseline characteristics | 24/2/2022 | Formal testing of baseline characteristics will not be performed. | Upon reviewer’s suggestion, and in line with CONSORT^**^ statement (Item 15). |
| Statistical analysis plan regarding efficacy outcomes | 24/2/2022 | Baseline measurement of the outcome will be included in the fixed part of the model. | Upon reviewer’s suggestion. |
| Statistical analysis plan regarding the 48-hour adverse events | 24/2/2022 | If substantial number of  48-hour adverse events are observed, mixed-effects logistic regression model will be performed on the proportion of infants with systemic adverse events separately or as a composite outcome. | Upon reviewer’s suggestion. |
| ^*^ No participant with this characteristic had been recruited till the time of this amendment; ^**^ Available from: https://www.bmj.com/content/340/bmj.c869  IPD: Individual Participant Data; ICMJE: International Committee of Medical Journal Editors | | | |
